# Group II Introns: Flexibility and Repurposing

**DOI:** 10.3389/fmolb.2022.916157

**Published:** 2022-07-05

**Authors:** Maria Costa

**Affiliations:** Université Paris-Saclay, CEA, CNRS, Institute for Integrative Biology of the Cell (I2BC), Gif-sur-Yvette, France

**Keywords:** group II intron ribozyme, self-splicing, intron-encoded reverse transcriptase, retrohoming, RNA structural rearrangement, targetron, spliceosomal pre-mRNA splicing, 3′-splice site selection

## Introduction

**FIGURE 1:**
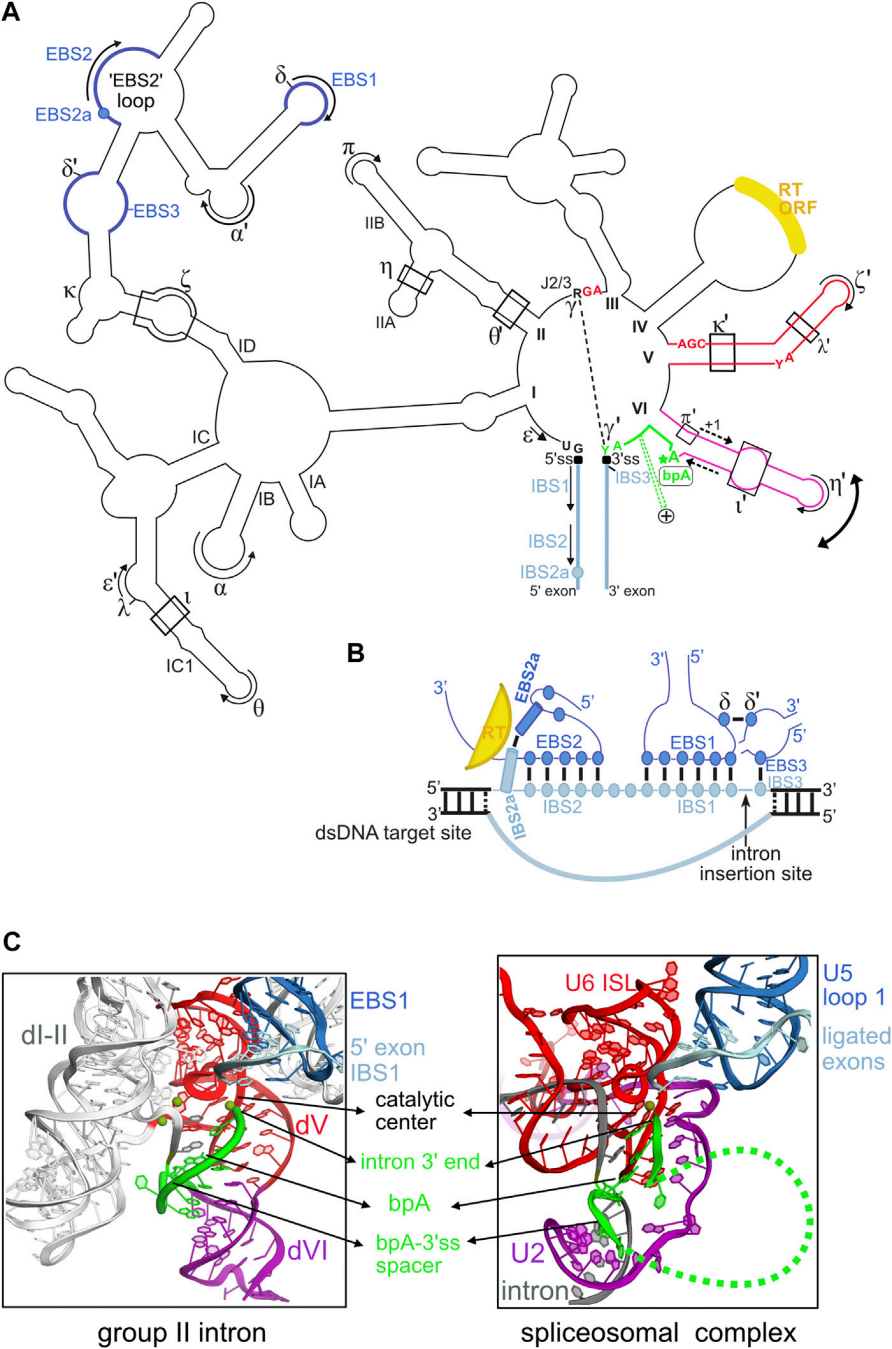
Structural plasticity of group II introns and its functional and evolutionary implications. **(A)** Outline of the secondary structure of a typical mitochondrial subgroup IIB1 intron. Only highly conserved intron nucleotides that are discussed in the manuscript are noted. Tertiary base-pairing interactions between the intron and its flanking exons are designated as (EBS-IBS) 1, 2, 2a and 3 pairings (see text). Long-range interactions involving intron sequences are designated by greek letters. The 5′ and 3′ splice junctions are indicated as black squares. The curved double arrow illustrates toggling of domain VI occurring between the two steps of splicing. The RNA tertiary interactions involved in this movement are: ι-ι′, which is specific to the first (branching) step, and η-η′ and π-π′, which are specific to the second (exon ligation) step. In addition, the intron-encoded RT also helps position domain VI for the branching reaction. The dashed-line arrows flanking the middle section of domain VI illustrate the strand shifting mechanism responsible for the alternate branchpoint-bulge conformations discussed in the text (only the 1-nt branchpoint conformation is depicted). The intron segment (“spacer”) from the branchpoint adenosine (bpA) to the 3’ splice site (3’ss), which is 7-nt long in these introns, is highlighted in green. The green dashed lines marked with a + symbol that emerge from this spacer illustrate the conserved insertions that can be found in this region in some introns, as discussed in the text. The reverse transcriptase ORF is always inserted in domain IV. **(B)** Diagram of the RNA-DNA interactions between IIB1 or IIB2 introns and their target sites highlighting the crucial role of the novel EBS2a-IBS2a base-pair in unwinding the DNA target duplex. The colors of the structural elements represented are consistent with the coloring of the secondary structure in **(A)**. Formation of EBS2a-IBS2a induces a “loop”around the EBS2-IBS2 pairing that should stabilize this small helix thus preventing re-association of the two DNA strands of the target. **(C)** Structural homology between the catalytic core of group II introns and the U2/U5/U6 snRNA catalytic core of the spliceosome bound to its intron substrate. Both catalytic cores are in their “second-step” conformation, immediately after exon ligation. The structure of the group II intron lariat shown was solved by X-ray crystallography at 3.5 Å resolution (PDB entry 5j02) and is color-coded according to the secondary structure in **(A)**. The catalytic center of group II introns is formed by a “catalytic triplex” involving highly conserved nucleotides of domain V and the J2/3 strand [these nucleotides are explicitly shown in red in panel **(A)**]. The catalytic triplex binds the two catalytic metal ions. The bpA-3’ss spacer, which is 7-nt long in the crystallized lariat, is highlighted in green. The spliceosomal 3D structure shown corresponds to the cryo-EM structure of the *Saccharomyces cerevisiae* post-catalytic P complex at 3.7Å (PDB entry 6EXN). Coloring of the RNA core is according to homology to group II intron RNA elements. Notably, the highly conserved internal stem loop (ISL) of U6 is homologous to group II intron domain V and positions of the invariant ACAGAGA box of U6 are homologous to positions of the J2/3 strand in group II introns. Indeed, the terminal GA of the ACAGAGA box interacts with the major groove of U2-U6 helix Ib to form a group II intron–like catalytic triplex that binds the two catalytic metal ions. The branch helix results from pairing of U2 snRNA (purple) to the branchpoint region of spliceosomal introns (gray), and the bpA-3’ss section is depicted in green. Note that only a few nucleotides of the bpA-3’ss spacer are visible in the cryo-EM structure, the green dashed line represents flexible regions of this spacer.

Group II introns are extraordinarily versatile self-splicing ribozymes and retrotransposable elements widespread in bacteria and in bacterial-derived organelles (mitochondria and chloroplasts) of fungi, algae, plants, and of some early-branching metazoans (Zimmerly and Semper, 2015). These large and highly structured ribozymes have a conserved secondary structure organized into six domains (I to VI) and they recognize the flanking 5′ and 3′ exons through extensive Watson-Crick base-pairings between the intron “EBS” (Exon Binding Sites) and the exon “IBS” (Intron Binding Sites) sequences (Figure 1A).

Despite their bacterial origin, it is now widely accepted that group II introns have been repurposed by natural evolution into the eukaryotic spliceosomal introns and the spliceosome (Toor et al., 2008; Costa et al., 2016; Galej et al., 2018). Both group II intron self-splicing and eukaryotic pre-mRNA splicing proceed through an identical pathway that involves two sequential transesterification reactions. First, a conserved “branchpoint” adenosine attacks the 5′ splice site generating a “lariat” intron intermediate with a 2′-5′ branch structure. During the second step, the terminal 3′OH group of the 5′ exon attacks the 3′ splice site leading to the ligation of the flanking exons and excision of the lariat intron.

In bacteria, most group II introns encode a multifunctional reverse transcriptase (RT) and invade genomes through “retrohoming”, a highly site-specific mobility process operated both by the ribozyme and its encoded RT enzyme (Lambowitz and Zimmerly, 2011). Retrohoming is initiated by “reverse splicing”, a pathway during which the excised intron lariat, in complex with its RT, catalyzes its own insertion directly into a specific DNA target containing the ligated-exons sequence (Zimmerly et al., 1995).

Natural group II introns are remarkably diverse regarding the structural organization of their ribozyme moiety, self-splicing pathways and the strategies they use to invade genomes. Here, I discuss recent findings regarding the splicing and mobility activities of group II introns that further emphasize the structural plasticity of these elements and their ability to be remodeled by molecular evolution into unique RNA-based machines, including the eukaryotic pre-mRNA splicing system.

## Remodeling of the Branchpoint-Carrying Domain VI and its Implications for 3′ Splice Site Selection and Alternative Splicing

Group II introns possess a single catalytic center that positions two metal ions responsible for catalysis of the transesterification reactions (Chanfreau and Jacquier, 1994; Toor et al., 2008; Marcia and Pyle, 2012; Costa et al., 2016). This organization implies that conformational rearrangements of the ribozyme must occur after the first step of splicing to remove the 2′-5′ branch out of the catalytic center and replace it by the 3′ splice site that will be cleaved during the second step.

The small terminal domain VI harboring the unpaired branchpoint adenosine (Figure 1A), has long been suspected to be dynamic and “step-specific” RNA tertiary interactions involving different sections of this domain have been demonstrated (Chanfreau and Jacquier, 1996; Li et al., 2011; Robart et al., 2014). On the other hand, the 3’ splice site is specified both by the γ-γ′ pairing, which involves the last intron nucleotide (Jacquier and Michel, 1990) and, in two of the three major intron subgroups, the EBS3-IBS3 pairing which constrains the first position of the 3′exon (Costa et al., 2000).

Recent crystal structures of an intron lariat captured just after splicing completion revealed the crucial role of the 2′-5′ branch structure in promoting proper assembling of the second-step active site (Costa et al., 2016). Unexpectedly, this work also brought to light a new mode of remodeling of domain VI that operates between the two steps of splicing. This novel mechanism consists in a rearrangement of the base-pairing pattern around the branchpoint adenosine. Domain VI remodeling likely occurs by strand shifting by one nucleotide and leads to alternate conformations at the branchpoint-bulge during the splicing pathway as demonstrated biochemically (Costa et al., 2016). Hence, the typical 1-nt (nucleotide) branchpoint-bulge conformation is necessary for the branching step, whereas the alternate, 2-nt bulge conformation, revealed by the crystal structures is required for stable docking of the 2′-5′ branch and accurate positioning of the 3′ splice junction into the catalytic center. Importantly, the published sequence analyses (Costa et al., 2016) reveal that introns with a 7-nt segment connecting the branchpoint adenosine to the 3′ splice site do have the potential to rearrange their domain VI secondary structure around the branchpoint bulge. These “7-nt segment” introns form the eukaryotic subgroup IIB1, bacterial subgroups B, E and some lineages of IIC. Only subgroup IIB2 introns appear to be unable to operate strand shifting around the branchpoint bulge despite the presence of a 7-nt segment.

More recently, cryo-electron microscopy (cryo-EM) structures of a bacterial subgroup IIB1 intron in complex with its reverse transcriptase and a DNA target allowed visualization of the large-scale toggling undertaken by domain VI between the two splicing steps (Haack et al., 2019). Interestingly, these structures did not show the alternate 2-nt branchpoint conformation demonstrated previously, a situation that is nevertheless in agreement with the crystallographic work (Costa et al., 2016) and could even be predicted based on the published sequence analyses mentioned above. Indeed, bacterial subgroup IIB1 introns have a 6-nt (instead of 7-nt) spacer, and this shorter distance between the branchpoint adenosine and the 3′ splice site likely obviates the need to remodel domain VI between the two steps of splicing. Accordingly, inspection of domain VI of bacterial subgroup IIA1 and IIB1 introns (Candales et al., 2012), RNAs which include a 6-nt spacer, suggests that the majority of these sequences lack the potential to stably rearrange the base-pairing pattern around the branchpoint bulge.

Even more interestingly and perhaps more profoundly, the previously unnoticed base-pairing rearrangement of domain VI also strongly suggests that the segment connecting the branchpoint adenosine to the 3′ splice site (hereafter referred to as the “bpA-3’ss spacer”) is much more flexible than previously recognized and this plasticity could have an impact on 3′ splice site selection. Interestingly, some natural group II introns that deviate from the typical 6-nt/7-nt length of the bpA-3′ss spacer support this hypothesis. A remarkable example is provided by a number of closely related IIB introns found in bacteria of the *Bacillus* genus that contain a large insertion of about 53/56 nucleotides (sometimes longer) located precisely between the end of domain VI and the intron 3′ splice site (Stabell et al., 2007, 2009; Figure 1A). These insertions fold into conserved stem-loop motifs and although their function remains a mystery, it is interesting to note that all these introns catalyze branching *in vitro* and the presence of the extra domain “VII” is important for efficient usage of the 3′ splice site (Tourasse et al., 2011). In another insightful example, the B. a.I2 intron, a IIB intron from *Bacillus anthracis*, self-splices not only at the expected 3′ splice site but also at an alternative 3′ splice site lying four nucleotides downstream the first one (Robart et al., 2004). Both 3′ splice sites are recognized by different sets of γ-γ′ and EBS3-IBS3 pairings and alternative splicing occurs both *in vivo* and *in vitro*. Interestingly, usage of the downstream 3′ splice site has crucial implications for genetic expression since it fuses two conserved ORFs and presumably drives translation of the downstream ORF. Hence, in this example the structural flexibility of the bpA-3′ss spacer appears to have been exploited by molecular evolution to create an alternative splicing system in bacteria.

## A Novel RT-Dependent Base-Pairing Interaction Between the Intron and its Target is Required for Retrohoming Into Double-Stranded DNA

The ability to directly invade DNA by reverse splicing and the high specificity of target recognition make mobile group II introns unique retrotransposons. The DNA target is primarily recognized by extensive arrays of Watson-Crick pairings (∼12–14 bp) between the intron EBS sites and the complementary IBS sequences lying on the top strand of the target (Figure 1B). Importantly, this recognition mode allows a given group II intron to be reprogrammed to insert into a desired DNA target by modifying the intron EBS sites to make them complementary to the IBS sites present in the target. This property has allowed biologists to repurpose group II introns into “targetrons” which are powerful RNA-guided systems with reprogrammable specificity for genetic engineering of bacterial genomes, independently from homologous recombination (Mohr et al., 2000; Karberg et al., 2001; Zhong et al., 2003; Perutka et al., 2004). The RT enzyme on the other hand, participates in DNA target binding by recognizing a small number of specific positions flanking the EBS-IBS pairings by mechanisms that remain largely unknown. During invasion of double-stranded DNA targets, the necessary unwinding of the double-helix is promoted by interactions between the mobile RNP and several positions lying on the 5′-distal section of the target site. The EBS2-IBS2 pairing is one of the players in the unwinding process (Singh and Lambowitz, 2001; Ichiyanagi et al., 2002; Coros et al., 2005). Using a combination of sequence analyses and genetic approaches, recent work has shown that ORF-carrying introns of structural subgroups IIB1 and IIB2 developed yet an additional base-pairing interaction between the intron lariat and its target that plays a crucial role in intron reverse splicing into double-stranded DNA (Monachello et al., 2021). This novel base-pair, named EBS2a-IBS2a, adopts a strict Watson-Crick geometry and induces an intricate architecture around the neighboring EBS2-IBS2 pairing that helps to maintain the open conformation of the DNA target helix (Figure 1B). Remarkably, the intron-encoded RT stabilizes the EBS2a-IBS2a interaction in a non-sequence-specific manner, which allows free exchange of all four Watson-Crick combinations (Monachello et al., 2021). Importantly, the DNA target position now identified as the “IBS2a” site was previously reported to be crucial for retrohoming of a subgroup IIB1 intron but its mechanism of recognition remained unknown (Zhuang et al., 2009), a situation that had constrained the intron-targeted genomic sites to harbor the wild-type base at this position. Elucidation of the rules governing recognition of IBS2a now makes it possible to “redirect” IIB1 and IIB2 introns to recognize any base found at the target site IBS2a simply by changing the nucleotide at the ribozyme EBS2a site according to base-complementary rules. Therefore, demonstration of the EBS2a-IBS2a pairing will broaden the application of the targetron technology by allowing the development of novel targetrons more extensively reprogrammable than previously thought.

## Discussion

The studies discussed above highlight the flexibility of group II introns and their ability to acquire novel structural features with specific functionalities in splicing and mobility. Over the course of evolution, this remarkable plasticity not only allowed the great diversification of group II introns, but it certainly played a key role in the molecular process that repurposed group II intron self-splicing into one of the most complex cellular function of eukaryotes: spliceosomal pre-mRNA splicing. It is hypothesized that during this evolutionary pathway, fragmentation of ancestral group II intron RNPs gave rise to the small nuclear RNAs (snRNAs) U2, U5 and U6 that constitute the active core of the extant spliceosome (Belhocine et al., 2008; Zimmerly and Semper, 2015). Concomitantly, domains of the intron-encoded RT playing a role in splicing were conserved in the essentiel spliceosomal factor Prp8 that forms the cavity housing the RNA-based active core of the spliceosome (Galej et al., 2013; Galej et al., 2018). Genetic and biochemical data accumulated over decades combined with the high-resolution structural data recently gathered by X-ray crystallography and cryo-EM fully support this scenario by revealing that the U2/U5/U6 snRNA core of the spliceosome bound to its intron substrate mimics precisely the catalytic core of group II introns. In particular, the architecture of the catalytic core in its second-step conformation is highly similar in both splicing systems (Figure 1C). Moreover, and as in group II introns, the 2′-5′ branch structure formed in spliceosomal introns participates in a network of RNA-RNA interactions that juxtapose the highly conserved 5′ and 3′ intron ends and promotes docking of the 3′ splice site into the catalytic center (Costa et al., 2016; Liu et al., 2017; Wilkinson et al., 2017). Interestingly, although the 2′-5′ branch structure was strictly conserved during evolution, the overall structure of the branchpoint-carrying domain VI was not. In fact, in the spliceosomal system the branch helix results from base-pairing of the branchpoint region present in all introns to a complementary region of U2 snRNA. Nevertheless, it is striking to observe that during pre-mRNA splicing, the spliceosomal branch helix undergoes the same large-scale movement (driven by spliceosomal factors) as domain VI, which implies evolutionary conservation of this essential toggling mechanism (Fica and Nagai, 2017; Haack et al., 2019). On the other hand, the typical 6-/7-nt bpA-3′ss spacer found in group II introns has not been evolutionarily conserved since in spliceosomal introns both the sequence and length of this spacer are highly variable (despite the presence of some conserved features such as the polypyrimide tract in metazoan U2-dependent introns). Nevertheless, the previously discussed flexibility of the bpA-3′ss spacer in group II introns may have been exploited during evolution of the eukaryotic spliceosomal system itself to develop mechanisms able to modulate 3′ splice site recognition. In agreement with this view, many spliceosomal introns in yeast form specific intramolecular secondary structures in the bpA-3′ss spacer region which are necessary for recognition of the proper 3′ splice site while masking illicit ones (Gahura et al., 2011). Interestingly, it has also been shown that some of these secondary structures mediate alternative 3′ splice site selection events (Meyer et al., 2011; Plass et al., 2012). Moreover, recent cryo-EM studies on the human spliceosome show that some of the metazoan-specific protein factors involved in alternative splicing interact with the bpA-3′ss spacer region at the exon ligation step likely influencing the anchoring of the 3′ splice site (Fica et al., 2019). Therefore, it is likely that during the course of evolution the bpA-3′ss spacer region has been independently repurposed several times to give rise to a variety of structural mechanisms capable of modulating 3′ splice site choice with profound implications for alternative splicing in eukaryotes.
